# Near-occlusion is difficult to diagnose with common carotid ultrasound methods

**DOI:** 10.1007/s00234-021-02687-x

**Published:** 2021-03-13

**Authors:** Elias Johansson, Davide Vanoli, Isa Bråten-Johansson, Lucy Law, Richard I Aviv, Allan J Fox

**Affiliations:** 1 Department of Clinical Science, Umeå University, Umeå, Sweden; 2 Wallenberg Center for Molecular Medicine (WCMM), Umeå University, Umeå, Sweden; 3 Department of Public Health and Clinical Medicine, Umeå University, Umeå, Sweden; 4 Department of Medical Imaging, Sunnybrook Health Science Center, University of Toronto, Toronto, Canada

**Keywords:** Carotid stenosis, Carotid near-occlusion, CT-angiography, Ultrasound

## Abstract

**Purpose:**

To assess the sensitivity and specificity of common carotid ultrasound method for carotid near-occlusion diagnosis.

**Methods:**

Five hundred forty-eight patients examined with both ultrasound and CTA within 30 days of each other were analyzed. CTA graded by near-occlusion experts was used as reference standard. Low flow velocity, unusual findings, and commonly used flow velocity parameters were analyzed.

**Results:**

One hundred three near-occlusions, 272 conventional ≥50% stenosis, 162 <50% stenosis, and 11 occlusions were included. Carotid ultrasound was 22% (95%CI 14–30%; 23/103) sensitive and 99% (95%CI 99–100%; 442/445) specific for near-occlusion diagnosis. Near-occlusions overlooked on ultrasound were found misdiagnosed as occlusions (*n* = 13, 13%), conventional ≥50% stenosis (*n* = 65, 63%) and < 50% stenosis (*n* = 2, 2%). No velocity parameter or combination of parameters could identify the 65 near-occlusions mistaken for conventional ≥50% stenoses with >75% sensitivity and specificity.

**Conclusion:**

Near-occlusion is difficult to diagnose with commonly used carotid ultrasound methods. Improved carotid ultrasound methods are needed if ultrasound is to retain its position as sole preoperative modality.

## Introduction

**Fig. 1:**
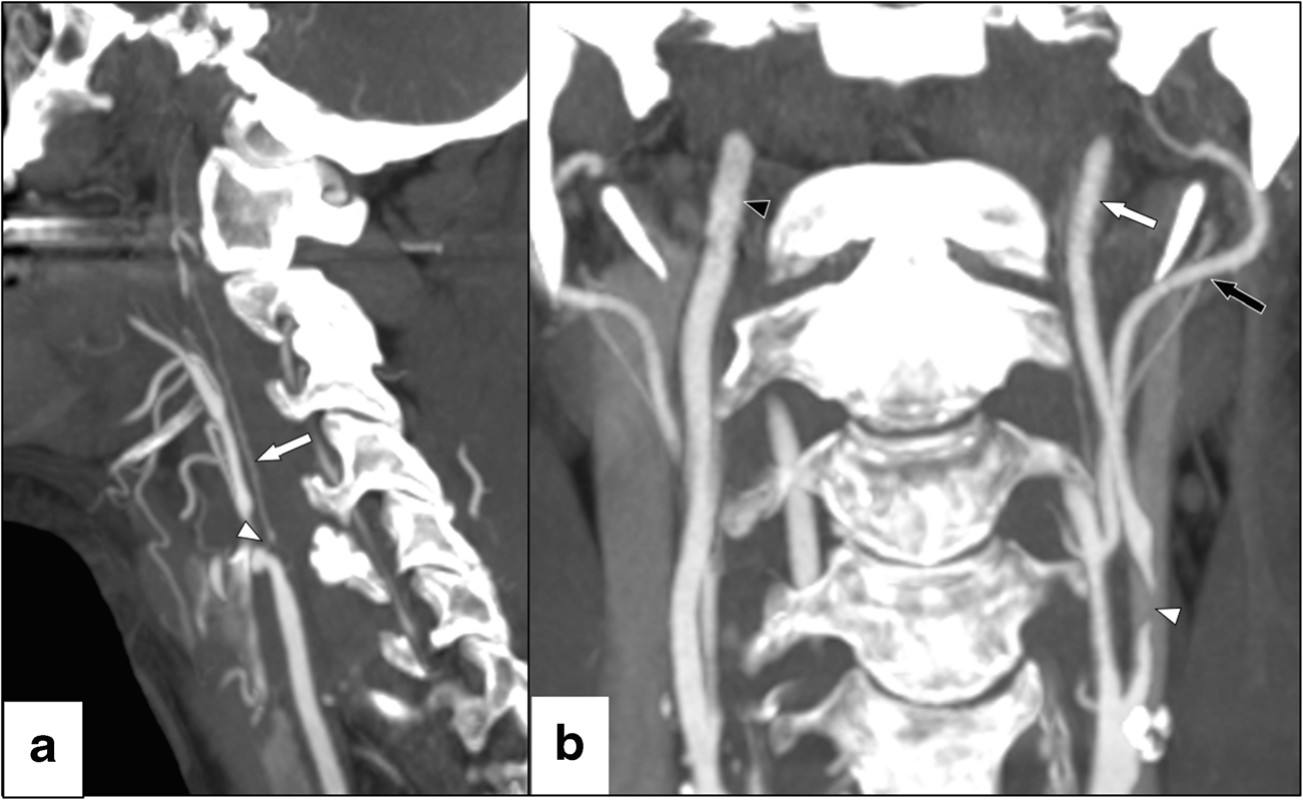
Two cases of left-sided near-occlusion. **a** Near-occlusion with full collapse, sagittal CTA. Beyond a severe stenosis (white arrowhead), the distal ICA is significantly size-reduced with a threadlike appearance. Distal ICA is 0.8 mm in diameter. **b** Near-occlusion without full collapse, coronal CTA. Beyond severe stenosis (white arrowhead), the distal ICA is normal-appearing but relatively small (white arrow, 2.8 mm diameter), smaller than contralateral distal ICA (black arrowhead, 3.9 mm diameter) and similar to ECA (black arrow, 2.4 mm diameter)

Carotid near-occlusion is a variant of severe carotid stenosis where, in contrast to conventional ≥50% carotid stenosis, the stenosis causes the artery to reduce in size (“collapse”) distal to the stenosis [1–3]. The collapse is considered a physiological response to reduction in pressure and flow. When the flow reduction is severe, the distal artery has a threadlike appearance (near-occlusion with full collapse, Fig. 1a). With moderate flow reduction, the distal artery often is “normal-appearing” albeit small (near-occlusion without full collapse, Fig. 1b) [1–3]. In contrast to symptomatic conventional ≥50% stenosis, the recent guideline recommendations for symptomatic near-occlusion is for conservative non-surgical management [4, 5], based on the muted effect of carotid endarterectomy for these patients in the pooled NASCET and ECST post hoc analysis [1].

**Fig. 2:**
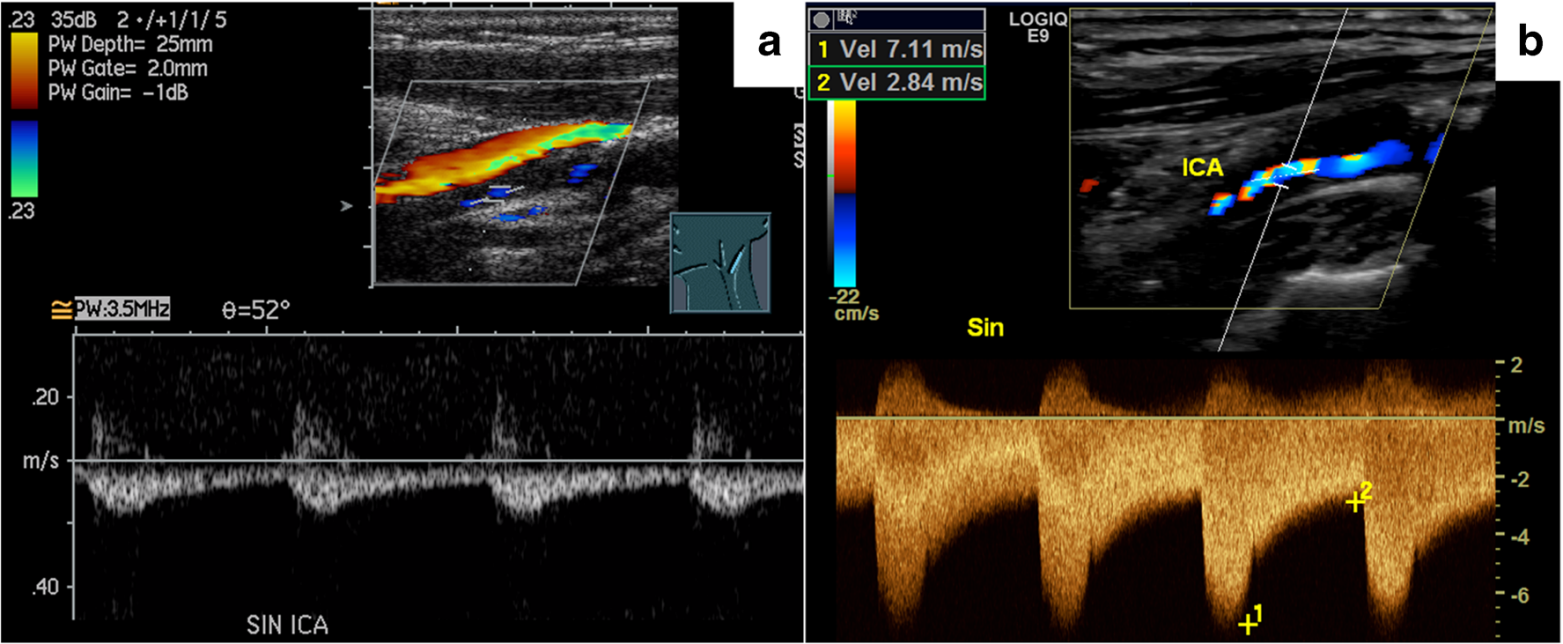
Ultrasound findings in two cases with near-occlusion. **a** Severe stenosis with low stenosis velocity, distinguishable from conventional stenosis by the low velocity. **b** Severe stenosis with high stenosis velocity, difficult to distinguish from conventional stenosis

Despite a large number of studies of carotid stenosis, the approach to diagnose near-occlusion with ultrasound remains unclear [6–11]. World Federation of Neurology ultrasound guidelines do not include near-occlusion as a category [6]. El-Saden studied 20 near-occlusions and found that 30% could be identified by low stenosis flow velocity or collapsed distal appearance [8]. Based on this small study, North American carotid stenosis grading guidelines state that ICA PSV in near-occlusion is “high, low or undetectable,” with only “low” being specific for near-occlusions (Fig. 2) [7]. Other studies have found that low flow velocity in the stenosis is very specific for near-occlusion [9, 10]. However, none of these studies specified what “low” velocity was [8–10]. El-Saden provided no threshold for collapsed distal appearance [8]. Khangure et al. recently defined “low” velocity as ICA PSV <125 cm/s as this threshold is considered to separate <50% stenosis from conventional ≥50% stenosis [11]. Khangure et al. found that only 7/46 (15%) of near-occlusions had this finding and that no commonly used velocity parameter was sensitive and specific for near-occlusion with high stenosis velocity [11]. However, Khangure et al. did not assess unusual findings that might indicate near-occlusion to the skilled observer (*suspected near-occlusion*) and was based on several databases [11]. None of these databases was dedicated to near-occlusion research [11].

The purpose of this study was to assess if commonly used ultrasound findings can be used to diagnose carotid near-occlusion.

## Materials and methods

We evaluated a dedicated retrospective near-occlusion research cohort from a single center including all cases that underwent CTA and ultrasound within 30 days of each other between January 2010 and December 2014. Cases underwent either ultrasound, CTA, or both based on clinical decisions, increasingly using both as routine. All ultrasound examinations were performed within the Department of Clinical Physiology at the University Hospital of Northern Sweden. Ultrasound exams of poor quality (e.g., demonstrating extensive shadowing) were excluded. Cases with revascularization between exams were excluded. CTAs were performed at or sent to the Department of Radiology at the University Hospital of Northern Sweden. CTAs were performed as part of clinical routine using various machines and various protocols at the University Hospital of Northern Sweden and 11 referring hospitals.

### CTA image interpretation

Near-occlusion was diagnosed when the distal extracranial ICA was reduced in size secondary to a proximal stenosis. Luminal reduction had to be visible as an extracranial ICA asymmetry [12]. Diagnosis was established using a systematic interpretive approach, weighing the information of four features of near-occlusion (stenosis severity, distal ICA size, distal ICA asymmetry, and ICA/ECA ratio), mirroring the approach in NASCET for CTA [1, 2, 12]. ICA/ECA ratio was calculated by dividing distal ICA diameter with ipsilateral ECA diameter just proximal to its terminal bifurcation, usually situated behind the jaw [12]. In cases of contralateral occlusion, ipsilateral near-occlusion diagnosis was considered when distal ICA was clearly size-reduced compared to ECA. Bilateral near-occlusion required bilateral clear size reduction compared to ECA. A conservative approach was used, only diagnosing near-occlusion when it was the most reasonable diagnosis. Asymmetric distal ICAs associated with asymmetric Circle of Willis were considered an important mimic to near-occlusion, as this is seen in 8% of persons without steno-occlusive disease [13]. Among the near-occlusions, full collapse was defined as a threadlike distal lumen, often smaller than the ECA (except when ECA was also reduced), whereas those without full collapse had a normal-appearing albeit small distal ICA [1–3]. Eight near-occlusions with full collapse with contrast visible distal to the stenosis but not yet reaching the skull base at the time of image capture were arbitrarily assigned a distal ICA diameter of 0.5 mm in the analyses. Occlusion was diagnosed when no contrast was visible beyond the lesion, accompanied by a rounded stump. In cases with a tiny residual lumen, where contrast opacity was reduced by technical limitations (e.g., partial volume effect), the luminal diameter was arbitrarily recorded as 0.5 mm for analyses. Among cases with conventional stenosis, the degree of stenosis was measured using established NASCET criteria [14].

The 4403 CTA exams (4042 patients) performed during the study period were re-evaluated by one observer (EJ, 5 years of carotid grading experience) [13]. Twenty-five cases had multiple eligible CTAs (<30 days to ultrasound without revascularization in between and acceptable exam quality). In 24, the exam closest to ultrasound was used. In one case, an earlier CTA was used even though a second CTA was closer to the ultrasound, because the second CTA revealed progression to symptomatic occlusion. All cases of suspected near-occlusion or near-occlusion mimics were re-evaluated in a blinded fashion by a second observer (AF, >40 years of carotid grading experience). Inter-rater disagreements were settled by a consensus discussion. Of the exams assessed by both observers, a random subset of 49 exams was assessed twice, for intra-rater reliability. All CTA reviews were performed blinded to ultrasound findings.

### Carotid ultrasound

Several experienced sonographers who were blinded to the CTA results performed the carotid ultrasound examinations as part of clinical routine. Specialists in clinical physiology (six in total) reviewed the images live and took additional images when needed. The same specialist signed the report. iU22 (Philips, Amsterdam, Netherlands), LogiqE9 (GE, Boston, USA), and S2000 and Acuson Sequoia (both Siemens, Munich, Germany) systems were used. A standard protocol was used, including visualization of the common carotid artery (CCA), ICA, and ECA in transverse and sagittal section, with and without color doppler. Pulsed doppler velocity image was routinely gathered in mid CCA and at the point of maximum stenosis, but not distal to the stenosis. Stenosis velocity was the main feature used for stenosis grading, not diameter measurement of the stenosis. Cases with no measurable flow were categorized as occlusion. Cases without detectable flow through the stenosis but with flow signal detectable beyond the stenosis with an ICA flow profile were categorized as *likely near-occlusion* (i.e., not likely occluded, as flow through the stenosis is assumed). For flow velocity analyses, *likely near-occlusions* were arbitrarily assigned stenosis PSV 10 cm/s and EDV 3 cm/s as there was an assumed flow, not zero flow. Two observers (IBJ and LL) retrospectively extracted PSV and end-diastolic velocity (EDV) data from stored images between them (no double extraction) for velocity analyses. Reported diagnosis, including cases with low PSV, was diagnosed as near-occlusion or <50% stenosis, and any mention of unusual findings that might suggest near-occlusion despite high stenosis PSV (*suspected near-occlusion*; i.e., possibly not a conventional stenosis) was noted by one observer (EJ). *Suspected near-occlusion* were cases where near-occlusion was at least suggested in the report despite high PSV, regardless of rationale. All ultrasound data extraction was blinded to CTA findings.

Since a threshold for “*low PSV*” is not well defined in the literature, we used both a *Low PSV* and *intermediate PSV* group in order to present as much data as possible. As *low PSV* should be specific for near-occlusion, to define it as lower than what constitutes a ≥50% stenosis is reasonable. To reproduce the methods of Khangure et al., “*Low PSV*” was defined as <125 cm/s, in turn based on that ≥125 cm/s is the threshold for ≥50% stenosis in two international consensus criteria [7, 15]. “*High PSV*” was defined as ≥145 cm/s as this was the locally used threshold defined for ≥50% stenosis based on published criteria [16]. The subsequent range in between, 125–144 cm/s, considered ≥50% by some, not others, was categorized as “*Intermediate PSV*.” The reported diagnosis was used to distinguish between <50% stenosis and near-occlusions among cases with *low* and *intermediate PSV.* To combine all approaches, “*Any indication of near-occlusion*” was defined as the combination of suspected near-occlusion, intermediate PSV, low PSV, and likely near-occlusion. Cases diagnosed as <50% stenosis in the ultrasound report was not considered to have *any indication of near-occlusion*.

Pulsatility index (PI) was calculated as (PSV-EDV)/mean velocity, where mean velocity was calculated as EDV+(PSV-EDV)/3 [11]. PSV and EDV ratios were calculated by dividing stenosis with CCA findings.

### Analysis and statistics

The side with the most relevant finding was analyzed. *Any indication of near-occlusion* on ultrasound was the main ultrasound outcome. Analyses of flow velocities were performed mainly to assess if flow velocity could be diagnostically useful among cases with high PSV without *any indication of near-occlusion*, but also to present flow velocities in all near-occlusions. Cases diagnosed as occlusion on ultrasound were excluded from flow velocity analyses.

We used mean, standard deviation (SD), 95% confidence intervals (95%CI), 2-sided *χ*^2^-test, *t*-test, and ANOVA using REGW-Q post hoc test, as specified. Receiver operating characteristic was used, analyzing area under the curve (AUC); diagnostic thresholds were set for maximal Youden index (where sum of sensitivity and specificity is maximum) and for maximal Hirsch-index (H-index, where sensitivity and specificity are as high and similar as possible), calculating resulting sensitivity, specificity, and positive and negative predictive values. We assessed all 28 possible combinations of two velocity parameters: For each combination, we first assessed a scatterplot to determine the reasonable range of thresholds, if values above or below the thresholds should indicate near-occlusion, and if “and” or “and/or” type of combination should be used. Then, all possible combinations of thresholds within the reasonable ranges were assessed to determine which combination produced the highest Youden index and H-index. *p* < 0.05 was pre-specified as border for statistical significance. SPSS 24.0 and Microsoft Excel were used in the calculations.

## Results

**Fig. 3:**
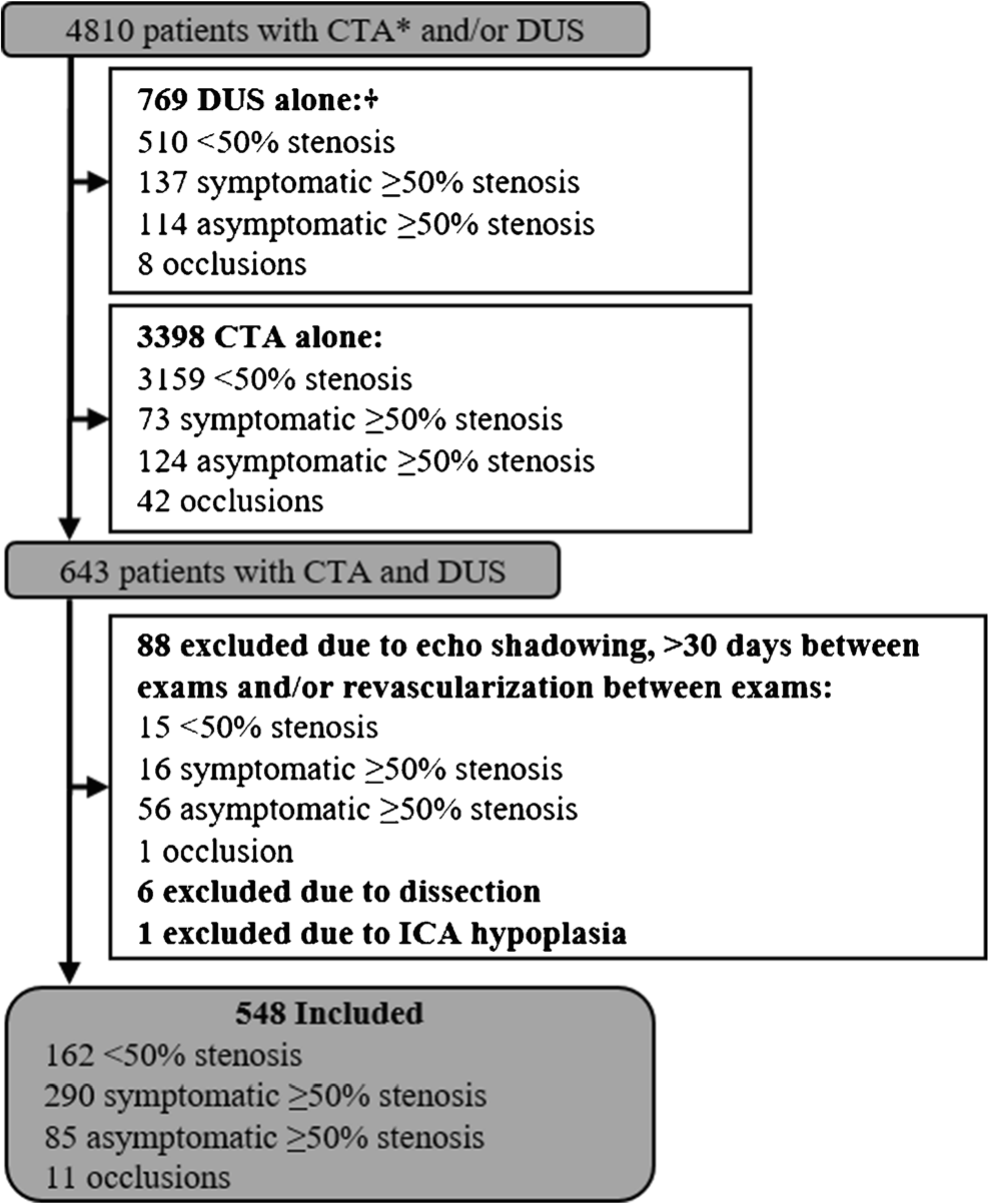
Study flow chart. *Excluding 37 exams with insufficient quality and one that could not be retrieved. ^†^Only cases without CTA (from any referral source) where DUS referral was from the stroke unit or case sent to stroke unit after identification of stenosis or occlusion on DUS

Five hundred forty-eight patients were included (Fig. 3). Mean age was higher in patients with conventional ≥50% stenosis than other stenosis groups, and occlusions tended to be less often symptomatic and tended to have longer delay between exams than other stenosis groups (Table 1). Large differences in CTA measurements were noted (Table 1). Of the 272 cases with conventional ≥50% stenosis, 30 (11%) had smaller ipsilateral distal ICA compared to contralateral ICA attributed to Circle of Willis variation (18 ipsilateral A1-hypo/aplasia, 4 contralateral large or fetal Pcom, 8 both).

**Table 1:** Baseline characteristics and CTA findings

Characteristic	<50% stenosis (*n* = 162)	Conventional ≥50% stenosis (*n* = 272)	Near-occlusion (*n* = 103)	Occlusion (*n* = 11)	*p*
Age years mean (SD)	68 (9)	72 (7)	69 (9)	65 (11)	<0.001^a,b^
Women n (%)	58 (36)	83 (31)	29 (28)	2 (18)	0.41^c^
Days between exams mean (SD)	5 (7)	5 (6)	4 (6)	10 (11)	0.07^a^
Symptomatic presentation^d^ *n* (%)	N/A	211 (78) ^d^	79 (77) ^d^	4 (36)	0.09^c,e^
CTA: Stenosis diameter mm mean (SD)	N/A	1.3 (0.4)	0.7 (0.2)	N/A	<0.001^f^
CTA: Distal ICA diameter mm mean (SD)	N/A	4.2 (0.6)	2.3 (0.9)	N/A	<0.001^f^
CTA: ICA ratio mean (SD)	N/A	0.97 (0.12)	0.52 (0.24)	N/A	<0.001^f^
CTA: ICA/ECA-ratio mean (SD)	N/A	1.64 (0.43)	0.81 (0.36)	N/A	<0.001^f^
CTA: Near-occlusion with full collapse *n* (%)	N/A	N/A	39 (38)	N/A	N/A

ICA ratio: ipsilateral/contralateral distal ICA diameter. ICA/ECA ratio: distal ipsilateral ICA/ipsilateral ECA. Stenosis diameter: smallest luminal diameter in the stenosis. N/A: not applicable.

^a^ANOVA

^b^On post hoc test, conventional ≥50% stenosis had higher mean age than all other groups at *p* = 0.02; with no difference between the other three groups (*p* = 0.54)

^c^2-sided *χ*^2^-test

^d^Symptomatic presentation: The stenosis/occlusion was ipsilateral to a recent ischemic cerebrovascular event, <50% stenoses are not considered symptomatic. Of the 92 cases with asymptomatic stenosis/occlusion, most (78%) were detected due to a suspected or confirmed cerebrovascular event (but did not have a recent ipsilateral ischemic event), 6% were incidental when examined for other diseases, 6% were follow-up exams of known asymptomatic stenosis, 5% were due to bruits, 4% were due to very late management of cerebrovascular event (>6 months after the event), 1% was found in research projects

^e^When comparing only conventional ≥50% stenosis and near-occlusion, no difference was seen (*p* = 0.89)

^f^*T*-test

**Fig. 4:**
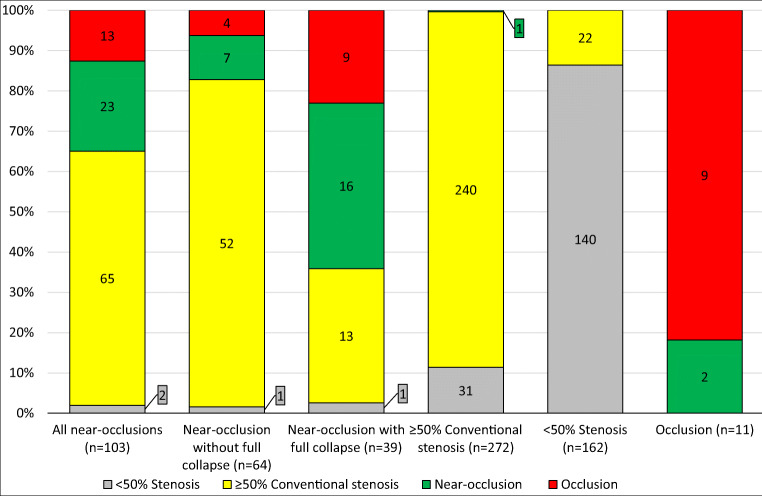
Comparison of CT-angiography (bars) and ultrasound findings (colors). Near-occlusion on ultrasound denotes *any indication of near-occlusion*, where two cases with <50% stenosis had *intermediate PSV* and diagnosed as <50% stenosis in the report

Twenty-two percent (95%CI 14–30%; 23/103) of near-occlusions had *any indication of near-occlusion*, more often among those with full collapse (41%; 95%CI 25–57%; 16/39) than those without full collapse (11%; 95%CI 3–19%; 7/64; *p* < 0.001, *χ*^*2*^) (Fig. 4). In those with full collapse, *low PSV* and *likely near-occlusion* both contributed to a large share of detected near-occlusion (both *n* = 6) (Table 2). In those without full collapse, all subsets of the combined outcome were similarly common (Table 2). Two near-occlusions (with and without full collapse each) had *intermediate PSV* (but none with *low PSV*) and were mistaken for <50% stenosis in the ultrasound report. Near-occlusions with full collapse were more often mistaken for occlusion than near-occlusions without full collapse (23%; 95%CI 9–37% and 6%; 95%CI 0–12%; respectively; *p* = 0.02, *χ*^2^). There were 3 false positive near-occlusions on ultrasound, 1 erroneously *suspected near-occlusion* that was a 54% stenosis on CTA and 2 *likely near-occlusion* that were occlusions on CTA. No case with conventional stenosis and *low or intermediate PSV* was mistaken for near-occlusion. Thus, *any indication of near-occlusion* was >99% specific (95%CI 99–100%; 442/445). When only cases with ≤7 days between ultrasound and CTA were considered, the sensitivity (20%; 95%CI 11–28%; 17/86) and specificity (99%, 95%CI 98–100%; 371/374) of ultrasound was similar to the outcome of the entire cohort.

**Table 2:** Types of near-occlusion findings on carotid ultrasound

Ultrasound finding	Near-occlusion without full collapse	Near-occlusions with full collapse	≥50% conventional stenosis	Occlusion
High PSV with suspected near-occlusion	3^a^	3^b^	1^c^	0
Intermediate PSV	1	1	0	0
Low PSV	2	6	0	0
Likely near-occlusion	1	6	0	2

All values are *n*. High PSV: stenosis PSV ≥145cm/s. Intermediate PSV: stenosis PSV 125–144 cm/s. Low PSV: stenosis PSV <125 cm/s. Likely near-occlusion: no flow detectable in the stenosis but noted in a low-flow artery with ICA profile beyond the stenosis

^a^Two by low distal velocity, one by mismatch between a tiny stenosis lumen on B-mode, but indication of moderate degree stenosis by stenosis PSV (307 cm/s)

^b^Two by mismatch between tiny stenosis lumen on B-mode, but indication of moderate degree stenosis by stenosis PSV (180 cm/s and 254 cm/s), one by very high stenosis PI (PSV 494 cm/s, EDV 16 cm/s, PI 2.73)

^c^Mismatch between a tiny stenosis lumen on B-mode, but indication of moderate degree stenosis by stenosis PSV (203 cm/s), CTA showed 54% stenosis

On group level, near-occlusions had higher mean stenosis PSV, stenosis EDV, PSV ratio and EDV ratio, and lower CCA PSV and CCA EDV than conventional ≥50% stenosis (Table 3). When only assessing the 65 near-occlusions with high stenosis PSV and without any indication of near-occlusion on ultrasound, these differences were similar or more pronounced (Table 3). No single ultrasound parameter had reasonable sensitivity and specificity to discriminate these 65 near-occlusions from conventional ≥50% stenosis. EDV ratio had the highest AUC (0.79) and Youden index, whereas stenosis EDV had the highest H-index (Table 4). Of the 28 possible combinations of parameters with adjusted thresholds, three combinations had both the highest H-index and Youden index, and were foremost based on EDV (Table 4). No parameter or combination of parameters was >75% sensitive and specific for near-occlusion.

**Table 3:** Group level differences in ultrasound parameters between conventional ≥50% stenosis, all near-occlusions and near-occlusions without *any indication of near-occlusion* on ultrasound (those currently missed). The 13 near-occlusion cases with occlusion finding on ultrasound excluded

Ultrasound parameter	Missing data	Conventional ≥50% stenosis (*n* = 272)	All near-occlusions (*n* = 90)	Near-occlusion without any indication of near-occlusion on ultrasound (*n* = 65)	*p*^a^ (*t*-test)	*p*^b^ (*t*-test)
Stenosis PSV	0	289 (138)	343 (197)	430 (141)	0.02	<0.001
Stenosis EDV	1	94 (68)	117 (94)	150 (87)	0.04	<0.001
CCA PSV	10	73 (25)	65 (20)	65 (19)	0.003	0.01
CCA EDV	10	15 (6)	10 (5)	10 (4)	<0.001	<0.001
Stenosis PI	1	1.32 (0.35)	1.33 (0.47)	1.26 (0.43)	0.80	0.27
CCA PI	10	1.73 (0.30)	1.95 (0.31)	1.91 (0.28)	<0.001	<0.001
PSV ratio	10	4.4 (2.8)	6.1 (4.5)	7.5 (4.3)	0.001	<0.001
EDV ratio^c^	11	7.7 (7.4)	13.7 (12.2)	16.4 (10.8)	<0.001	<0.001

All values are mean (SD). Stenosis PSV, Stenosis EDV, CCA PSV, and CCA EDV are in cm/s. PI = pulse difference/mean velocity, i.e., (PSV-EDV) / (EDV+((PSV-EDV)/3). PSV ratio = stenosis PSV / CCA PSV. EDV ratio = stenosis EDV / CCA EDV

^a^Conventional ≥50% stenosis compared with all near-occlusions

^b^Conventional ≥50% stenosis compared with near-occlusion without any indication of near-occlusion on ultrasound

^c^One case with 0 cm/s CCA EDV was arbitrarily set to EDV ratio 20

**Table 4:** Diagnostic analysis of discriminative ability of ultrasound parameters to separate conventional ≥50% stenosis and near-occlusions without any indication of near-occlusion on ultrasound (the subset currently missed). Cases with occlusion finding on ultrasound excluded. All single parameters and the three best combinations of parameters (out of 28) presented

Single parameters						
Parameter	AUC (95%CI)	Threshold	Sensitivity	Specificity	PPV	NPV
Stenosis PSV	0.76 (0.71–0.82)	≥305^a^ ≥361^b^	86 (78–95; 56/65) 71 (59–82; 46/65)	61 (55–67; 166/272) 71 (66–76; 193/272)	35 (27–42; 56/162) 37 (28–45; 46/125)	95 (92–98; 166/175) 91 (87–95; 193/212)
Stenosis EDV	0.71 (0.64–0.79)	≥114^a^ ≥110^b^	72 (61–83; 47/65) 72 (61–83; 47/65)	75 (70–80; 204/271) 74 (69–79; 201/271)	41 (32–50; 47/114) 40 (31–49; 47/117)	92 (88–96; 204/222) 92 (88–95; 201/219)
CCA PSV	0.57 (0.50–0.65)	≤93^a^ ≤68^b^	98 (95–100; 64/65) 54 (41–66; 35/65)	19 (14–23; 49/262) 55 (46–61; 144/262)	23 (18–28; 64/277) 23 (16–30; 35/153)	98 (94–100; 49/50) 83 (77–82; 144/174)
CCA EDV	0.70 (0.63–0.77)	≤9^a^ ≤12^b^	45 (35–57; 29/65) 68 (56–79; 44/65)	84 (79–88; 219/262) 60 (54–66; 156/262)	40 (29–52; 29/72) 29 (22–37; 44/150)	86 (82–90; 219/255) 88 (83–93; 156/177)
Stenosis PI	0.57 (0.48–0.65)	≤1.09^a^ ≤1.26^b^	42 (53–76; 27/65) 57 (45–69; 37/65)	74 (69–79; 200/271) 54 (48–60; 146/271)	28 (19–37; 27/98) 23 (16–29; 37/162)	84 (79–89; 200/238) 84 (78–89; 146/174)
CCA PI	0.67 (0.60–0.75)	≥1.87^a^ ≥1.87^b^	65 (53–76; 42/65) 65 (53–76; 42/65)	65 (59–71; 171/262) 65 (59–71; 171/262)	32 (24–40; 42/133) 32 (24–40; 42/133)	88 (84–93; 171/194) 88 (84–93; 171/194)
PSV ratio	0.76 (0.70–0.82)	≥5.0^a^ ≥5.3^b^	77 (66–87; 50/65) 71 (59–82; 46/65)	68 (63–74; 179/262) 70 (65–76; 184/262)	38 (29–46; 50/133) 37 (29–46; 46/124)	92 (88–96; 179/194) 91 (87–95; 184/203)
EDV ratio	0.79 (0.72–0.84)	≥6.2^a^ ≥9.0^b^	86 (78–95; 56/65) 72 (61–83; 47/65)	62 (56–68; 162/261) 72 (67–78; 188/261)	36 (29–44; 56/155) 39 (30–48; 47 /120)	95 (91–98; 162/171) 91 (87–95; 188/206)
3 best combination of parameters (out of 28)						
Parameters	Thresholds		Sensitivity	Specificity	PPV	NPV
Stenosis EDV and Stenosis PI	≥114 and/or ≥ 1.70^a^ ≥118 and/or ≥ 2.15^b^		89 (82–97; 58/65) 75 (65–86; 49/65)	63 (57–69; 171/271) 75 (70–80; 203/271)	37 (93–99; 58/158) 42 (33–51; 49/117)	96 (93–99; 171/178) 93 (89–96; 203/219)
CCA EDV and EDV Ratio	≤7 and/or ≥ 7.8^a^ ≤7 and/or ≥ 9.8^b^		83 (74–92; 54/65) 75 (65–86; 49/65)	69 (63–74; 179/261) 75 (70–80; 196/261)	40 (31–48; 54/136) 43 (34–52; 49/114)	94 (91–98; 179/190) 92 (89–96; 196/212)
Stenosis EDV and CCA EDV	≥114 and/or ≤ 7^a^ ≥110 and/or ≤ 4^b^		83 (74–92; 54/65) 74 (63–85; 48/65)	70 (64–75; 182/261) 74 (69–79; 193/261)	41 (32–49; 54/133) 41 (32–50; 48/116)	94 (91–98; 182/193) 92 (88–96; 193/210)

Data are % (95%CI; n/N). *AUC* area under the curve, *NPV* negative predictive value, *PPV* positive predictive value. Stenosis PSV, Stenosis EDV, CCA PSV, and CCA EDV are in cm/s

^a^Thresholds set at maximum Youden index

^b^Thresholds set at maximum H-index

Blinded inter-rater reliability for near-occlusion diagnosis on CTA was good: kappa 0.80 (95%CI 0.75–0.84). Intra-rater reliability was kappa 0.75 (95%CI 0.60–0.90) and 0.88 (95%CI 0.76–0.99) for each reviewer.

## Discussion

The main finding of this study was that most (63%) near-occlusions have high stenosis velocities without any clear difference from conventional stenosis in other commonly used parameters. Almost all commonly used velocity parameters were significantly higher or lower in this high-velocity near-occlusion compared with conventional ≥50% stenosis on group level. However, considerable overlap resulted in that no commonly used velocity parameters had >75% sensitivity and specificity, alone or in combination.

Near-occlusion is an angiographic diagnosis defined by artery size reduction beyond a severe stenosis [1–3]. Extent of distal ICA size reduction required for near-occlusion diagnosis has varied between reports. In studies where low stenosis velocity was relatively sensitive (71%) and very specific (99%) for near-occlusion, near-occlusion was defined as full collapse (“string sign”) alone [2, 9, 10]. We included both severe distal ICA collapses (with full collapse, sometimes called “string sign”) and subtle distal ICA collapses (without full collapse), similar to the pooled analysis for near-occlusion of NASCET and ECST [1]. Many clinicians do not pay attention to that 94% of near-occlusion in the pooled analysis of NASCET and ECST were near-occlusions without full collapse [1–3]. As this pooled analysis is the foundation of guideline recommendations [4, 5], the guideline recommendations are most applicable to near-occlusion without full collapse. Therefore, in studies of near-occlusion, those without full collapse should be included in the near-occlusion definition. However, this results in a reduced sensitivity of near-occlusion with carotid ultrasound.

With the expanded definition of near-occlusion, the sensitivity of commonly used ultrasound parameters for near-occlusion was low, but specificity was high, both in the current study and in Khangure et al., that also used this expanded definition [11]. Similarly, Palacios-Mendoza et al. recently confirmed that many near-occlusions are mistaken for conventional stenosis, but provided no velocity data or detailed definition [17]. We found a slightly higher sensitivity for the combined *any indication of near-occlusion* approach (22%) than Khangure et al. (15%) [11]. Khangure et al. only considered *low stenosis PSV* (<125 cm/s) and was unclear about how *likely near-occlusions* were analyzed [11]; the difference in sensitivity was accounted for by our findings in the *suspected near-occlusion* and *intermediate PSV* subset of *any indication of near-occlusion.* However, similar to Khangure et al., we found that no velocity parameter in the stenosis or CCA (i.e., those commonly used), alone or in combination, could differentiate between conventional ≥50% stenosis and the remaining near-occlusions with both sufficient sensitivity and specificity [11]. As many Youden index–based thresholds provide large differences between sensitivity and specificity, we added the H-index to clarify that the modest performance of ultrasound was not a result of poor choice of thresholds.

It remains unclear how to best detect near-occlusion on carotid ultrasound, although it is certain that improvements are needed. First, it is unclear what the best threshold for a “low stenosis velocity” should be. A relevant factor is that cases below this velocity threshold will be diagnosed as either near-occlusions or <50% stenosis based on visual inspection of the stenosis severity. We found that three cases were misclassified on such visual inspection in routine practice, two near-occlusions as <50% stenosis and one 54% stenosis as near-occlusion, highlighting that this approach is not without problems. Similar misdiagnosis by interpreting near-occlusion with low flow findings as <50% stenosis has been reported [11]. With these three misdiagnoses having stenosis PSV 133–203 cm/s, the best stenosis PSV threshold for “low velocity” is likely slightly above 125 cm/s, but dedicated prospective studies are needed to clarify this issue. Furthermore, *suspected near-occlusions* were not pre-specified categories, hence not systematically used in our entire cohort, some with similar findings might have been missed. All these findings have been reported previously [6, 9, 18], and additional types of unusual findings might be possible. Distal artery size was not systematically assessed, in large part due to the lack of diagnostic threshold for such a measurement [7, 8]. However, in our experience from angiography, the reduced artery size of many near-occlusions is modest (not clear unless comparing with other arteries) and only visible well beyond the bulb, a region commonly not reachable with ultrasound. Therefore, it is reasonable to suspect that assessment of distal artery size on ultrasound might not be very sensitive for near-occlusion. After this cohort was examined, low velocity distal to the stenosis has emerged as a possible new method specific for near-occlusion [18], i.e., not indicative for severe conventional stenosis as indicated in a guideline [6]. Also, ultrasound contrast might improve separation of occlusion and near-occlusion [19]. Thus, with improved definition of low flow, vigilance for unusual findings, and novel methods, better diagnostic outcomes for near-occlusion can likely be achieved. Therefore, our results are not indicative of best possible carotid ultrasound outcomes, but rather indicative of what many routine ultrasound laboratories are likely to currently achieve.

Ultrasound as the sole preoperative modality is accepted or recommended in guidelines [4, 5], which is supported by the findings of a systematic assessment and review [20, 21]. However, these assessments focused on discrimination of 50% and 70% stenosis and omitted near-occlusion, as did all their underlying ultrasound studies [20, 21]. Hence, when the community decided that ultrasound was reasonable as sole preoperative modality, the low sensitivity for near-occlusion with commonly used carotid ultrasound methods was not considered. As the difference between near-occlusion and conventional stenosis is management-altering according to current guidelines [4, 5], improvements in carotid ultrasound methods are needed if carotid ultrasound is to retain this prominent role. Also, the role of ultrasound might change in the future because recent studies reveal that some near-occlusions might benefit from revascularization [22–29].

Strengths of the study are relatively large size, dedicated near-occlusion data collection, and use of same interpretation approach and image interpreter for the angiographic comparison as the pooled analysis of the NASCET and ECST trials [1]. A limitation was that not all cases were examined with both ultrasound and CTA. Eleven cases had missing flow velocity data, mainly in the CCA, but all were in the control group of conventional stenosis. Although CTA, and not conventional digital subtraction angiography, was used as angiographic comparison, near-occlusions are well diagnosed on CTA [2, 3]. Our approach to combine different parameters should be interpreted with caution as it might have caused model-overfitting. That is, the results are, if anything, likely an overestimation of diagnostic performance. Therefore, the lack of clear diagnostic improvement with two common parameters compared to one common parameter or alternative threshold approaches makes it unlikely that any combination of these common parameters works well. Efforts to improve near-occlusion diagnostics should rather focus on assessing new parameters such as distal velocity and contrast use.

### Conclusions

Near-occlusion is difficult to diagnose with commonly used carotid ultrasound methods, foremost lacking in sensitivity. Improved carotid ultrasound methods are needed if ultrasound is to retain its position as sole preoperative modality.
